# Growth trajectories of prenatal embryos of the deep‐sea shark *Chlamydoselachus anguineus* (Chondrichthyes)

**DOI:** 10.1111/jfb.14352

**Published:** 2020-05-19

**Authors:** Faviel A. López‐Romero, Claudia Klimpfinger, Sho Tanaka, Jürgen Kriwet

**Affiliations:** ^1^ Department of Paleontology University of Vienna Vienna Austria; ^2^ School of Marine Science and Technology, Faculty of Marine Science and Technology Tokai University Shizuoka Shimizu‐ku Japan

**Keywords:** development, frilled shark, geometric morphometrics, jaw development, ontogeny

## Abstract

*Chlamydoselachus anguineus,* Garman 1884, commonly called the frilled shark, is a deep‐sea shark species occurring up to depths of 1300 m. It is assumed to represent an ancient morphotype of sharks (*e.g.*, terminal mouth opening, more than five gill slits) and thus is often considered to represent plesiomorphic traits for sharks. Therefore, its early ontogenetic developmental traits are important for understanding the evolution of its particular phenotype. Here, we established six stages for prenatal embryos and used linear measurements and geometric morphometrics to analyse changes in shape and size as well as their timing during different embryonic stages. Our results show a change in head shape and a relocation of the mouth opening at a late stage of development. We also detected a negative allometric growth of the head and especially the eye compared to the rest of the body and a sexual dimorphism in total body length, which differs from the known data for adults. A multivariate analysis of covariance shows a significant interaction of shape related to the logarithm of centroid size and developmental stage. Geometric morphometrics results indicate that the head shape changes as a covariate of body size while not accounting for differences between sexes. The growth pattern of stages 32 and 33 indicates a shift in head shape, thus highlighting the moment in development when the jaws start to elongate anteriorly to finally achieve the adult condition of terminal mouth opening rather than retaining the early embryonic subterminal position as is typical for sharks. Thus, the antero‐terminal mouth opening of the frilled shark has to be considered a derived feature.

## 1 INTRODUCTION

**FIGURE 1:**
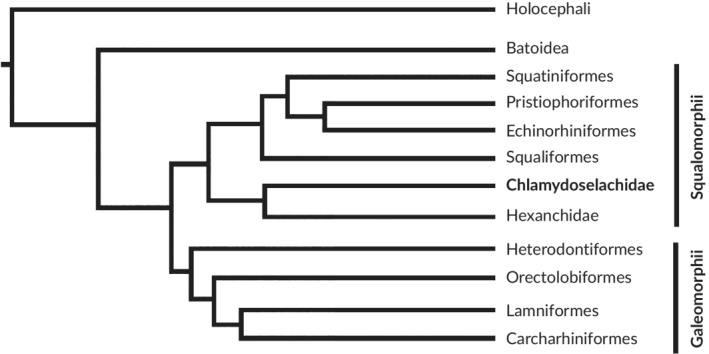
Phylogenetic position of *C. anguineus*, simplified after Amaral *et al*. (2017)

Samuel W. Garman described in 1884 an extraordinary shark, which he named the frilled shark, *Chlamydoselachus anguineus* Garman, 1884. Subsequently, Gudger and Smith (1933) described its anatomy in detail based on four adult females. The first description of its embryonic development was presented some years later by Gudger (1940). The frilled shark is assigned to its own family, the Chlamydoselachidae (Figure 1), which is believed to be the oldest living family of elasmobranchs (Garman, 1884; Gudger & Smith, 1933; Goto & Hashimoto, 1976; Goto, 1991; Tanaka *et al.,* 1990, 2013), although its affinities with Palaeozoic sharks was cautioned by Maisey and Wolfram (1984). The status of various morphological traits (plesiomorphic *versus* apomorphic) in this shark thus remains ambiguous. The embryonic development of elasmobranchs has become a major topic of focus for developmental biologists and also evolutionary palaeobiologists in recent years because it enables researchers to gain deeper insights into plesiomorphic and apomorphic morphologies and often mirrors the species' evolutionary path.

The habitat of the frilled shark is cold waters with a wide but discontinuous distribution where it occurs in water depths between 100 and 1300 m (Compagno, 1984; Shiobara *et al.,* 1987). It is considered a benthopelagic shark feeding on cephalopods, bony fish and even smaller sharks (Kirali *et al.,* 2000; Kubota *et al.,* 1991). It has an elongated, eel‐like body and it grows up to an average total length (TL) of 150 cm with a maximum reported TL of 196 cm (Gudger & Smith, 1933). Maturity is reached at a TL of 100 cm in males and 140 cm in females. Ebert and Compagno (2009) identified the populations around South Africa as separate species, *Chlamydoselachus africana*, that differs from the frilled shark in a smaller number of vertebrae, spiral valve and pectoral fin radial counts.

A prominent characteristic of the frilled shark is its elongated eel‐like body, which is very distinctive from other elasmobranch species. The head is dorso‐ventrally flattened, and its mouth is the most terminal positioned one in all shark species, except the whale shark *Rhincodon typus*, which is a filter‐feeder (Compagno, 1984). The frilled shark bears six gill slits including the first left and right gill slits, which are ventrally connected and form the eponymous frill (Smith, 1937). The number of gill slits is assumed to be a secondary increase in number since in most other shark species only five gill‐slits are present, and in the frilled shark the additional arch might have evolved independently from other hexanchoid sharks (Shirai, 1992).

Frilled sharks are aplacental viviparous with a year‐round mating and breeding season. Usually there are two to 10 embryos in each litter. They hatch within the mother's uterus when they reach a TL of 5.5 cm and remain there, feeding on their yolk sack until they are born at a TL of 40–60 cm (Tanaka *et al.,* 1990). The gestation period of *C. anguineus* is the longest period known for all vertebrates, lasting 3.5 years, and the growth rate of the embryos is around 10–17 mm per month (Tanaka *et al.,* 1990), although the gestation period estimation can be as low as 1–2 years (Compagno, 1984; Gudger & Smith, 1933).

The apparent ancient habitus, development and morphology of its teeth were considered in previous studies to better understand early evolutionary patterns in gnathostomes (Goto & Hashimoto, 1976; Goto, 1987; Smith *et al.,* 2018). However, developmental aspects of the external morphology that might provide information on character evolution in this and probably related taxa have not been considered up to now. The relationships between growth and changes in shape are relevant because they have an impact on the phenotypic diversity within species. Identifying the developmental moment when morphological change occurs is of utmost importance to comprehend how specific traits are established and how they vary among individuals. Studies of the ontogeny of morphology illustrate that developmental processes can help to identify constraints in morphological diversities as well as give examples for broader possible morphologies (Bolzan *et al.,* 2015; Cardini & Polly, 2013; Cheverud, 1982; Loy *et al.,* 1998; Openshaw & Keogh, 2014; Zelditch *et al.,* 2000). Despite these effects being more noticeable by comparing different but related species, there are other effects within the same species that could show how a defined shape is acquired. Some of the underlying processes in ontogenetic shape shift involve a decoupling caused by changes throughout the life cycle (Cvijanović *et al.,* 2014; Loy *et al.,* 1998; McGowan, 1988; Rose *et al.,* 2015; Strauss & Fuiman, 1985) or adaptations to particular feeding mechanisms or behaviours, both of which can influence the morphology of the individual (Bergmann & Motta, 2005; Bolzan *et al.,* 2015; Frédérich *et al.,* 2008; Genbrugge *et al.,* 2011; Meyer, 1990; Russo *et al.,* 2007). Particularly in elasmobranchs these patterns have been unambiguously observed and they contribute to the exploitation of possible resources throughout their life history (Dean *et al.,* 2007; Wilga *et al.,* 2016).

Earlier studies on the development of elasmobranchs have mainly focused on general descriptions of particular body parts and their anatomy (Balfour, 1881; El‐Toubi, 1952; Harrison, 1931; Holmgren, 1940; Jollie, 1971). However, only a few have attempted to present a staging system, which is useful for comparisons with other species (Ballard *et al.,* 1993; Castro & Wourms, 1993; Didier *et al.,* 1998; Rodda & Seymour, 2007; Maxwell *et al.,* 2008; Onimaru *et al.,* 2018). Most recently, studies have focused on post‐embryonic developmental traits (Fu *et al.,* 2016; Litherland *et al.,* 2009; Tomita *et al.,* 2018) and underlying developmental mechanisms (Adachi & Kuratani, 2012; Barry & Crow, 2017; Compagnucci *et al.,* 2013; Cooper *et al.,* 2017; Debiais‐Thibaud *et al.,* 2011; Eames *et al.,* 2007; Freitas *et al.,* 2007; Gillis *et al.,* 2012; O'Shaughnessy *et al.,* 2015). Because of their long generation time and reproduction mode, developmental studies of elasmobranchs are often difficult (Coolen *et al.,* 2008), and the diversity of descriptions on embryonic development consists of sporadic bycatches (Francis & Duffy, 2005; Gilmore *et al.,* 1983; Joung & Hsu, 2005; Tanaka *et al.,* 1990). Staging the development into sequences is fundamental to understand possible mechanisms operating at defined stages, which may have an impact on the phenotypic result. The goal of this study is to present new insights into the developmental changes of several morphological traits, especially the development of the anterior mouth opening in the frilled shark, which differs from the subterminal condition in most other sharks, to better understand the origins of its peculiar appearance.

## 2 MATERIALS AND METHODS

### 2.1 Ethical statement

The embryos used for the study were obtained from a survey conducted from 1981 to 1988 in the Suruga Bay, Japan, the samples from which are stored in the University of Shimizu, as described in Tanaka *et al*. (1990). Only individuals preserved in 70% ethanol were used for the analysis, and no tissue sampling or killing was involved in the present study.

### 2.2 Staging

Since there is no existing staging scheme for embryos of the frilled shark, we used different published information (see below) for comparison and to establish a staging sequence for the embryos. We also assumed that the embryos with the smallest body length are those from early developmental stages whereas the largest individuals are from later developmental stages.

We used 51 embryos (32 females, 19 males) from Suruga Bay, Japan. Forty‐three embryos were photographed using a Sony DSC‐H9 (Sony Corp., Tokyo, Japan) with a macro lens, whereas the remaining nine individuals were photographed using an Olympus E‐3 (Olympus Corp., Tokyo, Japan) camera with a 14–54 mm lens. All the photographs contain a scale next to the objects. Staging of the embryos was conducted by comparison of our individuals with descriptions and drawings from E.W. Gudger (1940) as well as the staging protocol of the lesser spotted dogfish *Scyliorhinus canicula* by Ballard *et al*. (1993) and the staging protocol of the brown‐banded bamboo shark *Chiloscyllium punctatum* by Onimaru *et al*. (2018). The lesser spotted dogfish is a galeomorph shark and spends parts of its lifetime in deeper waters too, which is why we chose it, and there is complete staging of the brown‐banded bamboo shark, which made it a useful comparison. The description of the staging of the chimaeroid fish *Callorhinchus milii* by Didier *et al*. (1998) was used for a comparison of the embryonic development of elasmobranchs with their sister group, the Holocephali, providing possibly another ancient morphotype. Accordingly, we identified categories from stage 31 up to stage 36. For each stage the following number of individuals was used: stage 31 (*n* = 1; female), stage 32 (*n* = 3; 2 females, 1 male), stage 33 (*n* = 8; 5 females, 3 males), stage 34 (*n* = 10; 9 females, 1 male), stage 35 (*n* = 14; 9 females, 5 males), stage 36 (*n* = 15; 6 females, 9 males).

### 2.3 Measurements

**FIGURE 2:**
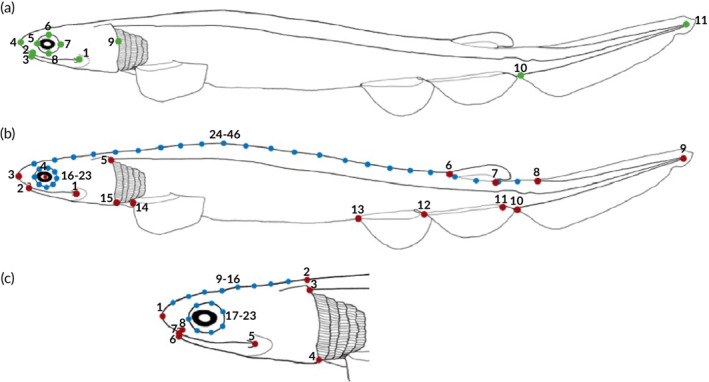
Position of the landmarks defined to estimate the variation in linear measurements and shape in *C. anguineus*. (a) Linear measurements landmarks (green dots): 1–2, upper jaw length; 1–3, lower jaw length; 4–9, head length; 5–7, eye diameter horizontal; 6–8, eye diameter vertical; 9–10, body length; 10–11, caudal fin length; 4–11, total length. (b) Body landmarks (red dots = landmarks; blue dots = semilandmarks): 1, labial cartilage articulation; 2, rostral edge of lower jaw; 3, rostrum; 4, eye center; 5, lateral line‐frill origin conjunction; 6, anterior insertion of dorsal fin; 7, posterior insertion of dorsal fin; 8, anterior insertion of caudal fin; 9, caudal fin posterior end; 10, anterior‐ventral insertion of caudal fin; 11, posterior insertion of anal fin; 12, anterior insertion of anal fin; 13, anterior insertion of pelvic fin; 14, anterior insertion of pectoral fin; 15, ventral origin of frill; 16–23, eye perimeter; 24–46, dorsal profile. (c) Head of *C. anguineus* embryo at stage 36 of development. The eight landmarks (red dots) and 15 semilandmarks (blue dots) are indicated in the corresponding anatomical positions: 1, rostrum; 2, posterior dorsal edge of head; 3, posterior dorsal side of frill; 4, posterior ventral side of frill; 5, labial cartilage articulation; 6, rostral edge of lower jaw; 7, rostral edge of upper jaw; 8, nostril; 9–16, dorsal craniofacial profile; 17–23, eye circumference

Linear measurements were defined by 11 landmarks set on each individual giving eight measuring distances (Figure 2a). The landmarks were chosen to be characteristic points, which can be identified easily in every picture. For the statistics and growth comparisons the TL was used as independent variable and values for body length (BL), head length (HL), caudal fin length (CF), upper jaw (UJ), lower jaw (LJ), and the diameter of the eye in vertical (EV) and horizontal (EH) plane were plotted and regressed to the predicted value using the package basicTrendline (Mei *et al.,* 2018) for R Cran 3.6.1 (2019).

### 2.4 Geometric morphometric analyses

A subset of 22 embryos out of the original sample of 51 specimens was chosen for subsequent analysis of shape with geometric morphometrics, since this subset presented no bending deformation that could lead to biased results in the shape. The number of individuals of each stage for this analysis were: stage 32 (*n* = 2), stage 33 (*n* = 4), stage 34 (*n* = 3), stage 35 (*n* = 4), stage 36 (*n* = 9). The shapes were defined according to two‐dimensional landmarks and semi‐landmarks, and the landmark coordinates were digitized using tpsDIG2 v.2.18 (Rohlf, 2002) (Figure 2b,c). The landmark coordinates were digitized twice on each individual and subject to a principal component analysis (PCA) to assess the accuracy of landmark capture.

Each subset of landmark coordinates was aligned by a generalized Procrustes superimposition as implemented in geomorph (v. 3.0.5) R package (Adams & Otarola‐Castillo, 2013). This method eliminates differences in size, position and orientation, and leaves only shape variation remaining for the analysis (Rohlf & Slice, 1990). Because each subset of landmark coordinates contains several semi‐landmarks the variation regarding its position along the curve was assessed considering the minimum bending energy between the target shape and the mean shape (Gunz and Mitteroecker, 2013). After superimposition, the Procrustes coordinates were used for a PCA to visualize the sources of variation in the specimens. Thin‐plate spline deformation grids were included to assist in illustrating the changes in shape from the mean along each PC axis as well as the minimum and maximum along those axes. The variation of head and body shape related to the size of the embryo was estimated by a multivariate analysis of covariance (MANCOVA) using the information of size as the logarithm of the centroid size (the square root of the sum of the squared distances of all the landmark coordinates), developmental stages, sex and the interactions between these variables. For statistical significance Goodall's F‐ratio and randomized residual permutation procedure (10,000 iterations) were used (Collyer *et al*., 2015). Significance in the interaction terms with (Shape ~ log(Csize)*Stage) and (Shape ~ log(Csize)*Sex) were interpreted as a difference in allometric trajectories between the stages or sexes of the individuals. A pairwise test for slope homogeneity was conducted with the advanced.procD.lm function in geomorph when the interactions were significant to assess the slope changes between developmental stages. Statistical significance rejecting the null hypothesis for a common slope was assessed by permutation using 10,000 iterations. The pairwise comparison of stage angle slopes was estimated in degrees. The shape change associated with the developmental stage was observed as a function of the regression of shape on the log‐transformed centroid size. The slopes of each developmental stage were plotted as the predicted shape scored against the log‐transformed centroid size.

## 3 RESULTS

### 3.1 Stages

#### 3.1.1 Stage 31

**FIGURE 3:**
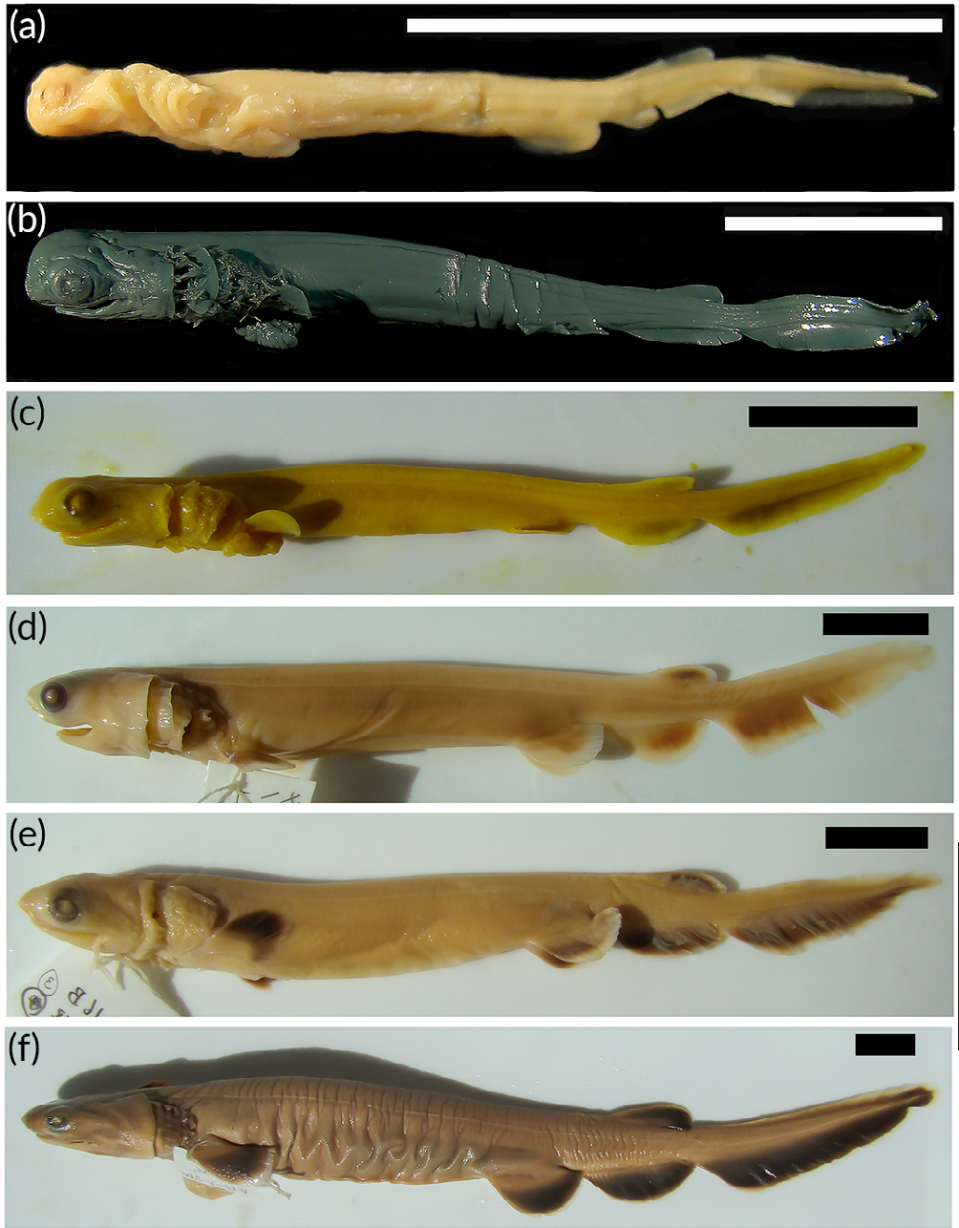
Fixed embryos of *C. anguineus* representing the developmental stages: (a) stage 31, (b) stage 32, (c) stage 33, (d) stage 34, (e) stage 35, and (f) stage 36. Scale bars: 20 mm

The LJ barely reaches the posterior end of the eye while the UJ extends to about the middle of the eye. The head shape is rounded, and pigmentation starts to surround the eye. The pectoral and pelvic fins are formed, with the pelvic fins being broader than the pectoral fins. No trace of the median fin fold is found in between the unpaired fins (Figure 3a). Embryos described by Gudger (1940) do not correspond to this size in morphology since the mouth of the described embryos are still in the diamond shape stage. The closer ones resembling the indicated stage are 32–34 mm

#### 3.1.2 Stage 32

The LJ starts protruding and the rostrum starts to change its position from its former ventral position to a more dorsal, anterior‐pointing position. Eye pigmentation becomes darker and surrounds the lens completely; still there is no pigmentation on the body. The pectoral and pelvic fins are longer than in the previous stage and the dorsal and anal fins are already well developed and separated from the caudal fin. All the unpaired fins start to show slight pigmentation along the margins. Small gill filaments are visible and there is a slight frilling on gills two to six (Figure 3b). Approximately corresponding to Gudger (1940) these embryos have a TL of 54–55 mm.

#### 3.1.3 Stage 33

The jaws are recognizably longer compared to the previous stage. The LJ extends anteriorly to the margin of the eye. The head is smaller and more compact, and the snout depicts a slightly pointed shape in a lateral view with the rostrum pointing anteriorly. The eyes are completely pigmented now, and the unpaired fins show an incipient dark pigmentation. Both paired fins (pectoral and pelvic) become longer and take in adult shapes, including an upcoming dark pigmentation on the margins. The caudal fin appears to turn upwards. There are no external gill filaments recognizable anymore. The lateral line starts to appear (Figure 3c). The embryos depicted by Gudger (1940) are heavily deformed; the closest one described appears to correspond to the 66 mm embryo.

#### 3.1.4 Stage 34

Both jaws are extending forward, surpassing eye level, and meanwhile have nearly the same length. The snout is now blunter and points straight forward. The body becomes pigmented with a brownish shade. Pectoral and pelvic fins elongate and become broader, the lower caudal lobe already shows the adult form. The body, between pectoral and pelvic fins, becomes more elongated (Figure 3d). The embryo described by Gudger (1940) at 103 mm could correspond in morphology to this stage.

#### 3.1.5 Stage 35

The LJ almost reaches its terminal position. The snout is pointed in lateral view and the nasal capsules are in their final positions. The bases of the paired fins are well developed and the dorsal and anal fins are already similar to the ones seen in adults. The lateral line system is more defined than in the stages before but not completely developed yet. Frills are occurring on the first gill (Figure 3e). The embryo at 124 mm by Gudger (1940) is the most similar.

#### 3.1.6 Stage 36

The mouth opening now reaches its final terminal position. The head is slightly dorso‐ventrally compressed and the snout is more prominent. The body pigmentation is now complete and more intense than before with the fins (both paired and unpaired) being darker than the body. Paired fins now also reach their adult shape and the unpaired fins have grown a little broader to reach final shape, including the appearance of the terminal notch in the caudal fin. Frills are well visible and detected on all gills and the lateral line is clearly defined (Figure 3f). The embryos from 185 to 240 mm described by Gudger (1940) correspond to this stage.

### 3.2 Analysis of linear measurements

**FIGURE 4:**
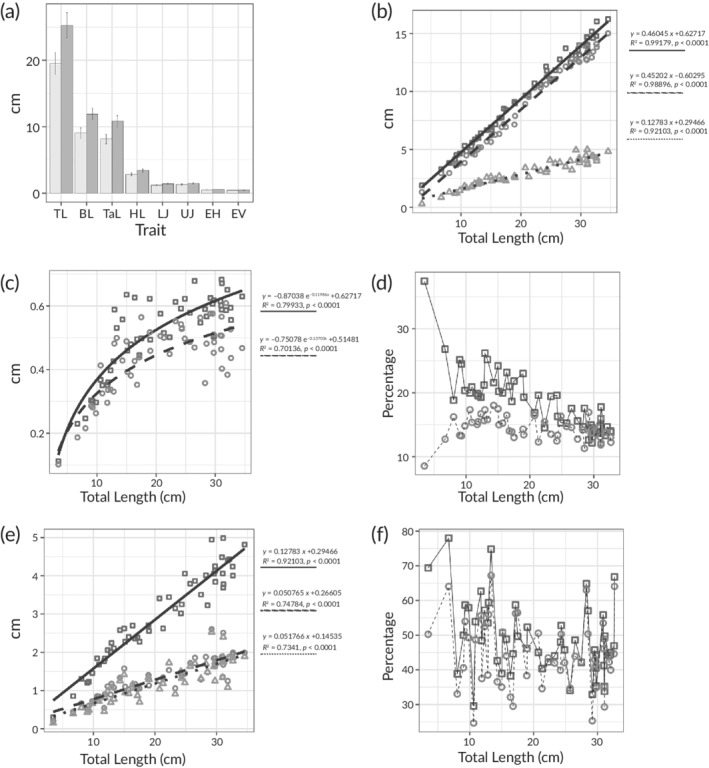
Linear measurement analysis of the embryos of *C. anguineus*. (a) Averages with standard error bars for each of the linear measurements from the landmarks described in Figure 2a Sex (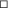) Female, (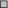) Male. (b) Relation of the BL, CF and HL as a variable of the total length. (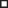) BL, (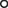) CF, (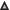) HL. (c) Eye length in both horizontal and vertical diameter. (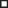) EH, (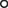) EV. (d) Proportion changes of the eye diameter and head length through development. (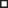) EH‐%HL, (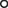) HL‐%TL. (e) Length growth of the head, and upper and lower jaw. (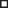) HL, (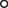) UJ, (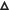) LJ. (f) Growth of the upper and lower jaw as percentage of the head length. (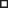) UJ‐%HL, (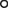) LJ‐%HL. BL, body length; CF, caudal fin length; HL, head length; EH, eye diameter horizontal; EV, eye diameter vertical; EH‐%HL, eye diameter horizontal percentage of head length; HL‐%TL, head length percentage of total length; UJ, upper jaw length; LJ, lower jaw length; UJ‐%HL, upper jaw percentage of head length; LJ‐%HL, lower jaw percentage of head length

The 51 measured embryos generally show an allometric growth pattern in most of the measured distances. Starting with the means of the TL we have to acknowledge that there are more females than males in this sample with the smallest and the biggest individual both being females. Still, males are significantly bigger than the females (*t*‐test, *P* = 0.03) (Figure 4a). The same applies to many measured distances, which undergo an elongation during development (*e.g*., BL and CF) (Figure 4a). Analysing the distribution of lengths between head (defined by LM 4 and 9), body (defined by LM 9 and 10) and tail (defined by LM 10 and 11) it is noticeable that the head is the shortest body part, which also elongates the least.

The body, understood as everything between head and tail, is the longest part with the highest tendency to further growth (Figure 4b), and grows proportionally at the same rate as the CF, while the HL also keeps growing but at a lower rate compared to the BL and CF. Comparing the EV to the EH, a slight elliptical shape on the horizontal axis can be noticed, which might increase in postnatal development (Figure 4c). When considering the EH as a percentage of HL, it is noticeable that the eye in relation to the head decreases in size while the head continues to grow in relation to the TL (Figure 4d).

Analysing the growth rate of the mouth opening in connection with the growth of the head it seems that the jaws grow at a relatively slower rate compared to the head. There is a visible difference between the UJ and the LJ at all times, with the UJ being always longer than the LJ, resulting in a constant gap. These differences, however, are not noticeable in the proportion of the UJ and LJ to the HL as a function of the TL (Figure 4e). On average the UJ is 0.1 cm longer than the LJ, but this is not significant (*P* > 0.5). Both jaws appear to grow little through the measured developmental stages. Furthermore, a convergence of the jaw as a percentage of the head is discernible in both parts of the jaw (Figure 4f). This would lead, according to the linear trend, to closure of the gap between UJ and LJs, with both of them reaching the same length.

### 3.3 Shape variation of body and head

**FIGURE 5:**
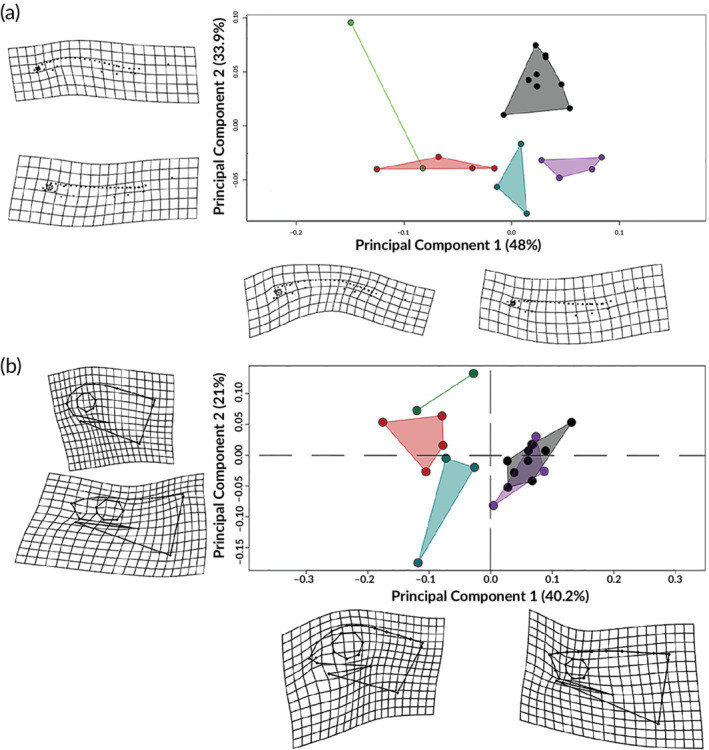
PCA of the body and head shape variation in *C. anguineus*. (a) Body shape variation due to deformation effects by the preservation of the individuals. (b) Head shape variation among frilled shark embryos through development. Colours indicate the developmental stage: (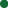) stage 32; (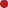) stage 33; (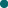) stage 34; (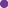) stage 35; (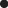) stage 36

Estimating the ratio of the mean squares of the individuals and replicates from a nested ANOVA showed a repeatability of 0.97. A discriminant function analysis on the replicate measurements after a PCA revealed that each capture session could not be assigned as different groups (*P* = 0.5) (Supporting Information Figure S1). The PCA shows that for both the body and the head configuration the PC1 and PC2 account for over 81.9% and 62.7%, respectively, of the total variation (Figure 5). For the body shape, the PC1 is related to the dorsal curvature and the upwards position of the caudal fin tip. However, this shape change might be related to the bending effect of preservation. Nevertheless, the positive values of PC1 shows that the later stages (34, 35 and 36) are grouped in this area, while the earliest stages share the negative side of PC1 (Figure 5a). The Unbend function in tpsUtil was tried in an attempt to eliminate the deformation effect, but without success. PC2 also describes shape changes similar as in PC1. In this case the deformation effects also seem to be part of the preservation bending, with some differences between developmental stages. Earlier stages seem to have a more straightened dorsal side of the body, which might tend to curve as development proceeds. Since these effects could not be removed from the analysis, we focused mainly on the head shape configuration for further analysis.

The head shape along PC1 describes a more rounded craniofacial profile on the negative values (Figure 5b), a longer distance from the origin of the maxilla to the preorbital region, and the postorbital region of the head appears to be elongated as development progresses. The UJ and LJ also elongate. In the earlier stages, the anterior tips are positioned slightly at the middle of the eye, and as development continues the tips extend anteriorly to surpass the anterior margin of the orbit. The shift in the elongation of the jaws is first recognizable with the UJ. At earlier stages, the UJ starts to protrude along with the rostrum while the LJ only reaches the terminal position at around stages 34 and 35. Both jaw tips are at the same terminal position at stage 36.

The orbit also appears to be larger in earlier developmental stages in relation to the BL, but appears to slow its growth in the later stages. Later shape changes during development become more apparent in a more straightened frontonasal profile with the tip of the rostrum turning upwards and also a longer postorbital region. PC2 describes the position of the eye from the middle to a more dorsal location and a more flattened head shape, although this is not reflected as a shape change related to a particular developmental stage. The head shape was not sufficient to determine sexual dimorphism among the individuals analysed (Supporting Information Figure S2). However, since the sampling size for each stage does not include both sexes for all the developmental stages, the morphospace might not be accurate. Perhaps the inclusion of more male specimens for the earlier stages might provide a better picture to investigate possible sexual dimorphism patterns in the future.

### 3.4 Head allometry variation

**FIGURE 6:**
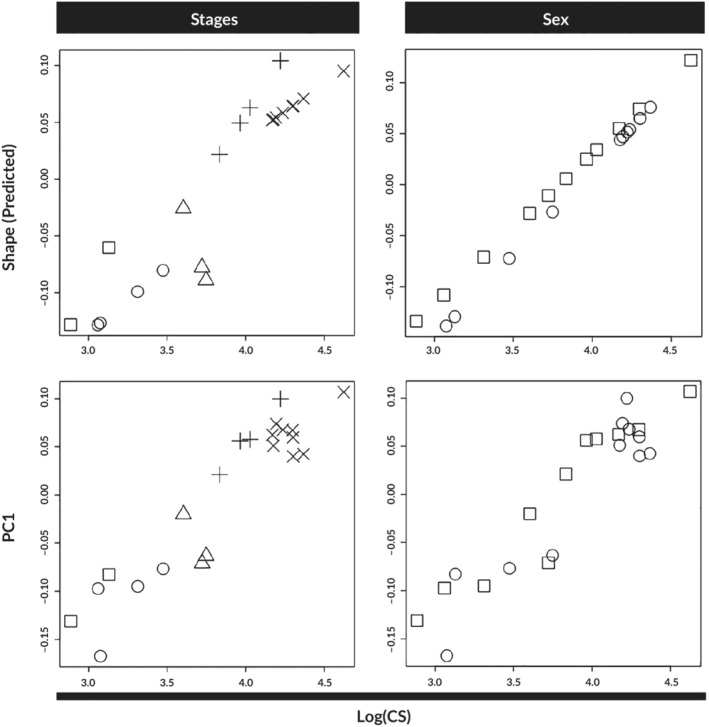
Allometric slopes and shape variation of the head of *C. anguineus* as a function of logarithm of centroid size. Both developmental stages and sex are compared. (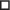) stage 32; (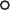) stage 33; (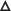) stage 34; (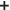) stage 35; (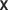) stage 36; (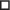) female; (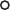) male

**FIGURE 7:**
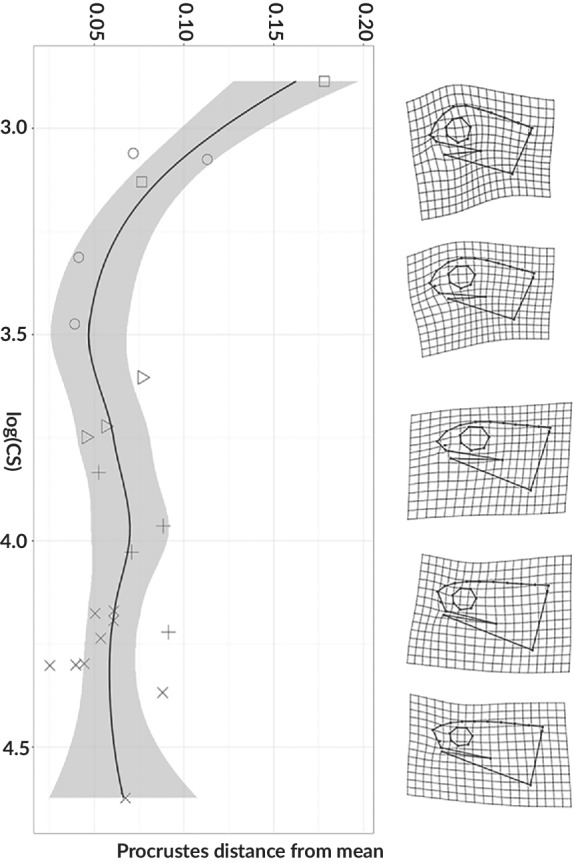
Shape divergence of the head of *C. anguineus* as a function of Procrustes distance from mean to the logarithm of centroid size (solid line; grey shading = 95% confidence interval) of the head shape through the development. (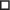) stage 32; (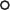) stage 33; (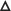) stage 34; (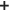) stage 35; (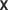) stage 36

Since there were differences in the head shape related to developmental stage, a MANCOVA was performed to examine interactions between shape, size, stage and sex (Table 1). The head shape differences are mainly explained by size (*r* ^2^ = 0.397) and developmental stage (*r* ^2^ = 0.162). The significant interactions of size and developmental stage in both configurations suggest that allometric slopes might differ between developmental stages. When we compared this covariation of head shape assuming the sex as an important factor and its interaction to the centroid size, we observed that the interaction is slightly significant (Table 1). The allometric slope plots show that some developmental stages differ in the intercept and that the intercept of later stages takes place in the upward *y* axis (Figure 6). After the confirmation of significant interactions between size and developmental stages and also sex (Table 1), a pairwise slope test was conducted to reveal divergence points during the development of the head shape or if there was an effect of sexual dimorphism related to size and shape. The results suggest that the earliest stages are the ones where the shape starts to shift (stages 32 and 33), and we noticed changes in most traits of the head, like the upward shift of the rostrum and the onset of jaw protrusion. However, there were no differences between the sexes and probably other characters could be more useful in the compared stages (Table 2). Taking the Procrustes distances from the mean configuration of the individuals plotted against the PC1 scores revealed that the earlier stages are the ones with the highest variation in shape, and as development progresses the later stages tend to converge towards the mean shape and their variation is reduced overall (Figure 7).

**TABLE 1:** Covariance of shape and size related to ontogenetic changes (stages) and sex of the individuals

Head shape	Df	SS	MS	*R* ^2^	*F*	*P*
log (Csize)	1	0.20022	0.200216	0.39715	17.2306	**0.0001**
Stage	4	0.08204	0.020511	0.16274	1.7651	**0.0002**
log (Csize):Stage	4	0.08244	0.020611	0.16353	1.7737	**0.0001**
Residuals	12	0.13944	0.011620			
Total	21	0.50414				
log (Csize)	1	0.12443	0.124434	0.31974	9.7884	**0.0001**
Sex	1	0.01469	0.014686	0.03774	1.1553	0.1165
log(Csize):Sex	1	0.02123	0.021229	0.05455	1.6699	**0.0201**
Residuals	18	0.22882	0.012712			
Total	21	0.38917				

*Note*. Df, degrees of freedom; SS, sum of squares; MS, mean squares; *R* ^2^, coefficient of determination; *F*, *F* ratio; *P*, *P* value. Bold indicates significant *P* values.

**TABLE 2:** Pair‐wise *post hoc* test of allometric slope angles between stages and sexes of the individuals

	Stage 32	Stage 33	Stage 34	Stage 35	Stage 36
Stage 32	**–**	***0.0138***	0.6098	0.3613	***0.0082***
Stage 33	79.75450	**–**	0.1869	0.2159	0.9095
Stage 34	130.76903	101.16381	**–**	0.3618	0.1973
Stage 35	81.77449	108.39498	108.2021	**–**	0.1835
Stage 36	62.84690	83.97788	114.0396	81.93991	**–**

*Note*. Upper half (cut diagonally) indicates *P* values and lower half indicates angles. Significant *P* values reported in bold.

## 4 DISCUSSION

The characters selected for the definition of the developmental stages follow the definitions of morphological‐based landmarks rather than a time‐ or length‐based system. This is due to the variable gestation periods between several elasmobranch species (Ballard *et al.,* 1993; Didier *et al.,* 1998; Maxwell *et al.,* 2008; Rodda & Seymour, 2007), with the frilled shark having probably the longest gestation period of up to 3.5 years (Tanaka *et al.,* 1990). Overall the stages were well defined and subsequent analyses reflected the grouping of the individuals belonging to their respective stage. Since it is assumed that the frilled sharks represent a basal elasmobranch group, the introduction of a staging scheme and the subsequent description of the development of specific traits is of particular interest to understand the origin of its peculiar morphology. Some of the most noticeable changes are related to the development of the jaws and the rostrum. While almost all other elasmobranch species display a ventrally, subterminally located mouth opening, which reaches its final position at a quite early stage [around stage 28 in catsharks and bamboo sharks (Ballard *et al.,* 1993; Onimaru *et al.,* 2018)], the rather terminal positioned mouth opening in the frilled shark starts to move to its future position at stages 31–32 and reaches the final position near the end of the embryonic development. In the winter skate *Leucoraja ocellata*, Maxwell *et al*. (2008) point out that the change in the position of the jaws starts at an even earlier stage (stage 27) and that this shift in timing can be related to the specialized dorso‐ventrally flattened body shape.

Other characters such as the paired and unpaired fins seem to be more variable throughout development. We were not able to detect a median fin fold from which the unpaired fins develop (Mabee *et al.,* 2002). However, it is expected that by the time of the formation of the pectoral and pelvic fins this structure might already be absent (Witten & Huysseune, 2007). Several studies point at specific events during ontogeny where certain shape changes occur and are central for particular adaptations (Genbrugge *et al.,* 2011; McGowan, 1988; Meyer, 1990; Rose, 2009; Russo *et al.,* 2007). We observed in our sample a larger shape variation in the early stages and we were able to estimate the moment of morphologic convergence to one defined specific morphology. Finally, the staging of the frilled shark embryos will allow further comparisons with other elasmobranch species at similar stages to improve the understanding of character evolution.

Sexually related dimorphism in body size is known in several groups of vertebrates (Adkins‐Regan & Reeve, 2014; Arak, 1988; da Silva *et al.,* 2017; Francis & Duffy, 2005; Malmgren and Thollesson, 1999; Monnet & Cherry, 2002; Rogers *et al.,* 2017; Woolbright, 1983). Elasmobranchs tend to display sexual dimorphism in size (Francis & Duffy, 2005; Rogers *et al.,* 2017; Rolim *et al.,* 2015; Semba *et al.,* 2011) and even in specific characters like the mouth shape of the small‐spotted catshark, which is, for example, longer and narrower in males than females (Ellis and Shackley, 1995). In some species, like the Caribbean sharpnose shark *Rhizoprionodon porosus* or the smalltail shark *Carcharhinus porosus*, several measurements of head, body and fins indicate that males are generally larger than females (Barbosa Martins *et al.,* 2015). In the case of the frilled shark linear measurements of adults (adulthood being defined by sexual maturity) indicate that females, conversely, are generally larger than the males without this difference in size being particularly noticeable at birth (Tanaka *et al.,* 1990). Both sexes still experience the same shape changes during development, which is displayed by the nonsignificant difference between their allometric slopes.

The linear measurements in our sample show that the males are significantly larger than the females even though there was a larger number of females in the sample, including the largest and smallest individuals. This discrepancy in total BL between the embryonic or newly born female and the sexually mature one leads us to the suggestion that females either grow faster or that they grow over a longer period of time before reaching sexual maturity. As a trend, deep‐sea elasmobranch species have lower growth rates, greater longevity and late age at maturity (Rigby & Simpfendorfer, 2013). In a paper by Girard and Du Buit (1999) a comparison of the growth rate differences between sexes of two viviparous deep sea shark species – the Portuguese dogfish *Centroscymnus coelolepis* and the leafscale gulper shark *Centrophorus squamosus* – was drawn and showed that the females were larger in TL in both embryonic stages and as adults. From our samples we cannot observe this difference clearly since at later stages the sample size is not large enough to display such trends.

The eye diameter was measured vertically and horizontally and showed only a very small amount of growth in both distances from the smallest to the largest embryo, reaching a plateau, which might continue its growth at a slower pace throughout the life of the individuals. Litherland *et al*. (2009) showed that eye growth differs significantly between the sandbar shark *Carcharhinus plumbeus*, which inhabits coastal waters, and the shortspine spurdog *Squalus mitsukurii*, which inhabits deeper waters of the continental shelf. In their paper Litherland *et al*. (2009) suggest that the growth through lifetime takes place due to constant adaptations to the current surrounding of the individual. Perhaps the eyes of the frilled shark present this pattern since there is only little light in the water depths at which they live. The eye size of the frilled shark, in percentage of the head of recently hatched individuals of different size, could provide more support on its possible adaptations.

The jaws elongate quite constantly with the growth of the head, as shown by their length measurements. The constant difference of UJ and LJ (the LJ being the shorter one) leads to an observable gap, which can be detected in every stage and is also known from adult individuals (Smith, 1967). From our results, the loss of the gap can neither be observed in late embryonic stages nor in adults and is probably a deviation due to skewed effect. Nonetheless a shrinking of the size of the gap between the two jaws is detectable during development, displayed in lateral view.

The craniofacial traits are of particular interest because many species have an observable trend that helps to explain the developmental basis of particular adaptations according to the development of the craniofacial traits (Ahi, 2016; Eames & Schneider, 2008; Hall *et al.,* 2014). Morphological changes within the same elasmobranch species previously were detected by functional analysis of the feeding apparatus. The displayed shifts in individuals were related to a change of diet during ontogeny, which also involved a change in size and bite performance of the feeding apparatus (Fahle and Thomason, 2008; Fu *et al.,* 2016; Huber *et al.,* 2008; Lowry & Motta, 2008; Wilga *et al.,* 2016). In some cases, similar morphologies of different species seemingly evolved multiple times independently (Dean *et al.,* 2007). However, most of these changes occur during postnatal development and reflect trends the juveniles experience while growing up. While analysing the jaw growth of the frilled shark we noticed that the shape changes and elongation of the jaws occur later in embryonic development, compared with other elasmobranch species, and continue up to the latest stage we analysed. Although the frilled shark has been positioned as a primitive form of elasmobranch (Goto & Hashimoto, 1976), several studies suggest that some of its characters (especially the jaw suspension and number of gill slits) could be derived (Amaral *et al.,* 2017; Bustamante *et al.,* 2016; Soares and de Carvalho, 2013; Tanaka *et al.,* 2013). The developmental sequence of jaws in other species could shed light on the changes we observed, since – as pointed out by Maxwell *et al*. (2008) in *Leucoraja ocellata* – some of these derived features can occur at earlier stages, as in the case of the jaws. It is noteworthy, however, that the jaws start to develop in a subterminal position as is typical for most sharks, but reach a terminal position late in development through anterior elongation of the jaws.

The caudal fin is another interesting structure as it appears not to change its shape at all throughout the stages we analysed. It has been reported that the caudal fin shape changes in other species like the tiger shark, *Galeocerdo cuvier*, the great white shark, *Carcharodon carcharias*, and the spiny dogfish, *Squalus acanthias* (Fu *et al.,* 2016; Reiss & Bonnan, 2010; Tomita *et al.,* 2018), which might reflect a shift in function towards their apex predator niche or possibly a biomechanical response to swimming mode. We found that in the frilled shark the caudal fin seems to experience only very little change in shape. However, since our sample comprises prenatal individuals only, it is possible that the caudal fin experiences shape changes after birth. Therefore, we cannot assess if certain prenatal shape constraints are related to deep‐sea adaptations for sure or if there are changes in postnatal development to give the frilled shark its specialized phenotype.

## 5 CONCLUSIONS

We present for the first time a comparison of the embryos of the aplacental viviparous deep‐sea frilled shark through several stages, which serves as a reference point for further studies among other elasmobranch species. The characters we analysed highlight the changes that the frilled shark experience in prenatal ontogeny. Although the changes appear to be remarkable, it should be kept in mind that the gestation period in this species is one of the longest known. Nevertheless, by using the size as a proxy of developmental time, we can estimate that overall some traits change relatively fast while others change at a slower pace. The highly variable shape in earlier stages and the subsequent convergence towards the mean shape is prominent and suggests a tendency to reduce its variability when the organism approaches a terminal stage in development. Regarding the sexual dimorphism, a difference in the mean TL was detected that differs from the size dimorphism in mature individuals. This character might display growth variation within postnatal individuals and might continue throughout the lifespan of this shark. Finally, a set of traits that could be useful to determine a general adaptation to deep‐sea environments is difficult to assess, but further studies on eye development might provide new insights. Furthermore, we provide additional evidence that suggests that the terminal position of the jaws in elasmobranchs could be a derived trait rather than a plesiomorphic condition as is generally assumed.

## AUTHORS' CONTRIBUTIONS

F.A.L.‐R., C.K. and J.K. conceived/designed the study. C.K. and F.A.L.‐R. collected the raw data and performed the analyses. F.A.L.‐R., C.K., J.K. and S.T. wrote the manuscript. All authors gave final approval for publication.

## Supporting information


**SUPPORTING INFORMATION FIGURE S1** **AF1_Supp_Replication.pdf** Morphospace of the landmarks replicates to test the repeatability of the coordinates capture

**SUPPORTING INFORMATION FIGURE S2** **AF2_Supp_Morphospace_sex.pdf** Morphospace of the shape variation between sexes (female = red; male = black). (a) Body shape and (b) head shape
